# Assessing the Impacts of Relative Wealth and Geospatial Factors on Water Access in Rural Nepal: A Community Case Study

**DOI:** 10.3390/ijerph17186517

**Published:** 2020-09-07

**Authors:** Naseeha Islam, Pramesh Koju, Reetu Manandhar, Sudip Shrestha, Charlotte Smith

**Affiliations:** 1 School of Public Health, University of California, Berkeley, CA 94720, USA; naseehaislam@berkeley.edu; 2 Department of Community Programs, Dhulikhel Hospital—Kathmandu University Hospital, Dhulikhel 45200, Nepal; kojupramesh@gmail.com (P.K.); reemanandhar@gmail.com (R.M.); sudip@kusms.edu.np (S.S.)

**Keywords:** WASH, wealth inequality, geospatial information systems, suitability analysis

## Abstract

As one of the poorest nations, citizens of Nepal lack access to safe, affordable, and sufficient drinking water. While many nationwide studies have been performed at a country or regional level in Nepal to determine regions of the highest vulnerability, this study uniquely recognizes the economic heterogeneity within a single rural village and assesses the impact of household socioeconomic status on water access at the intracommunity level. Household surveys in a rural village setting provided the information for a locally-informed relative wealth index. A spatial analysis determined suitable locations for future installation of improved water sources to prioritize water access for the community’s most vulnerable households. Three sites were shown to be optimal for future water source construction. This study provides a blueprint to assess water inequalities within a single village and incorporate forward-thinking development approaches to water access.

## 1. Introduction

Water, sanitation, and hygiene (WASH) are fundamental for human health and quality of life on both the individual and societal levels. However, globally 663 million people, predominantly in the global south, still lack adequate access to reliable water supplies [1]. In 2015, the United Nations (UN) published the 17 Sustainable Development Goals (SDGs) for 2030 as a more people-centered, expanded version of its Millennium Development Goals established in 2000 [2]. The SDGs focus the importance of WASH as a facet of international progress and development by incorporating “Clean Water and Sanitation” as its sixth primary goal [3]. The World Health Organization (WHO) defines water access in accordance with the SDGs, stating basic water access is having a water source within 1 km or a 30 min round trip; yet millions around the globe do not meet this international standard for basic access [4,5].

Lack of access to water has many well-known impacts at the individual level. The most common disease associated with a lack of clean water sources is diarrheal disease from enteric pathogens such as *E. coli* and *E. hystolitica* [6,7]. Other known morbidities include stunting and wasting [8], premature birth, and low infant birth weight [9]. Moreover, the provision of basic access to improved water sources has a profound impact on the social dynamics of certain populations, influencing education rates, gender equality, and intergenerational economic development [10,11]. Globally, there is a distinct accessibility difference between urban and rural populations, with rural regions often facing more neglect from development programs [12] and having less improved water sources overall [13]. This urban–rural divide is compounded by differences in water accessibility on the basis of socioeconomic status. Wealth is known to be a significant confounder in studies investigating water access, with access increasing with increasing wealth [9,14,15]. It is clear that WASH is deeply rooted in environmental justice, and limiting these inequities should be a focus when considering issues of water accessibility.

Nepal is an extremely diverse nation, consisting of multiple ethnicities, cultures, and languages, along with having considerable differences in population densities, topography, natural resources, and weather patterns [16]. A landlocked country of nearly 30 million people, it is one of the poorest and least developed nations in the world [17]. Its international status as one of the most slowly developing countries demands attention toward its WASH status in the face of the 2015 Sustainable Development Goals. Nepal is considered a greatly water-insecure nation, likely exacerbated by its complex geophysical landscape and high regional variation [18].

In the last two decades, the Nepali government has made significant strides toward improving the WASH status in communities throughout the nation. However, progress has predominantly been concentrated within more urban areas of the country. As 80% of the Nepali population is known to reside in rural regions, this urban prioritization of WASH leaves the majority of the country’s people out of primary consideration. Shrestha et al. reported that 25% of survey respondents residing in rural districts had no adequate sanitation infrastructure, and over 50% of the respondents claimed to have limited access to suitable water [19]. In alignment with global trends, the rural–urban divide and resulting socioeconomic disparities in Nepal contribute to WASH-based inequalities. Nationwide cross-sectional studies have indicated that it is the nation’s poorest communities, primarily found in the rural periphery of the country, who face the greatest barriers to water access [20].

Socioeconomic status is a significant indicator for water accessibility, with the poorest people being at higher risk for reduced access to improved sources. However, these studies have been primarily conducted at the national level with respect to the wide heterogeneity of socioeconomic status across village, district, and city lines with little attention to the diversity of wealth and accessibility even within specific rural communities. While the value of larger cross-sectional studies cannot be ignored, it is equally important to recognize the inherent heterogeneities that exist within single communities. This study aimed to bolster community empowerment, which could often be lost in broader studies. An intention of this study was to work with the community to document local information that could then be used to assess and address the results of local interventions. Moreover, very few studies have incorporated direct research-based advice for reasonable solutions that can be implemented within a relatively short time frame, once again empowering the particular community at hand to take control of the well-being of its own members. For these reasons, we determined whether there is a relationship between relative household wealth and access to improved water sources, to assess WASH inequalities at the intra-community level.

## 2. Materials and Methods

### 2.1. Data Collection

From 2016, students from the organization GlobeMed at Berkeley have been in a committed, collaborative partnership with the Department of Community Programs (DCP) of Dhulikhel Hospital, an adjunct of Kathmandu University in Nepal. The action-oriented partnership is focused on developing sustainable WASH practices and resources to the rural regions of Nepal through the department’s 18 outreach centers. Specifically, students of GlobeMed at Berkeley have been involved in the development projects of the Bolde outreach village in the Kathmandu valley. In June of 2018, data were collected by members of the DCP from 72 school children in grades 6–8 at the two secondary schools in Bolde. Data included in this 2018 exploratory analysis included anthropometric measurements and stool samples in order to determine the impacts of unimproved water and inadequate nutrition on individual health. These measures and samples were analyzed by the DCP and contributed to the secondary data analysis reported here.

In June of 2019, surveys were conducted at village households to assess family-level contributions to water access, sanitation, and hygiene in Bolde. We included the same cohort of students, which includes the entire village population of grades 7–9 (*n* = 72), for the sake of consistency with prior research and familiarity with the Department of Community Programs. As this study was performed in a small rural community, our convenience sample accounted for over 15% of the study population (*n* = 470 households). All 72 of the students who were included in the survey study lived in different households and were within walking distance of the school they attended. Informed consent was gained from all participating household representatives in the study by the DCP. Consent was also given by secondary school principals to interact with students and their families on school grounds as necessary.

Survey answers were collected using handheld tablets with the software CommCare (Dimagi, Cambridge, MA, USA) [21] due to the ubiquity of CommCare within Nepali research teams, including those at Dhulikhel Hospital, thus making it an ideal software program in terms of its ease of use and translatability to local programs. The handheld tablets simplified location tracking and made it possible to immediately download respondent answers into a central server.

There were two data collection teams, each consisting of two GlobeMed student interns and one hired data collector/translator. Prior to data collection, the teams practiced collaborative rehearsal in order to ensure cross-surveyor consistency and reduce interviewer bias. Both groups met with secondary school principals to confirm the location of the students’ households. Because many households were spread far apart and walking was the only means of transportation, households were organized into local clusters to maximize the number of household visits that could be completed each day. No maps were available in such a rural village region, so travel to each household was reliant upon the general directions indicated by the principals and based on the word of mouth of community members. Upon arrival at the household, data collection teams obtained consent and proceeded with the survey (conducted with the child’s mother or another caregiver if the mother was not available). The data collectors conversed with the household caregivers in Nepali to obtain survey answers, while student interns (upon earning consent) took notes and observations about the water sources, building materials, and hygiene products present in the visible vicinity.

### 2.2. Data Analysis

**Figure 1:**
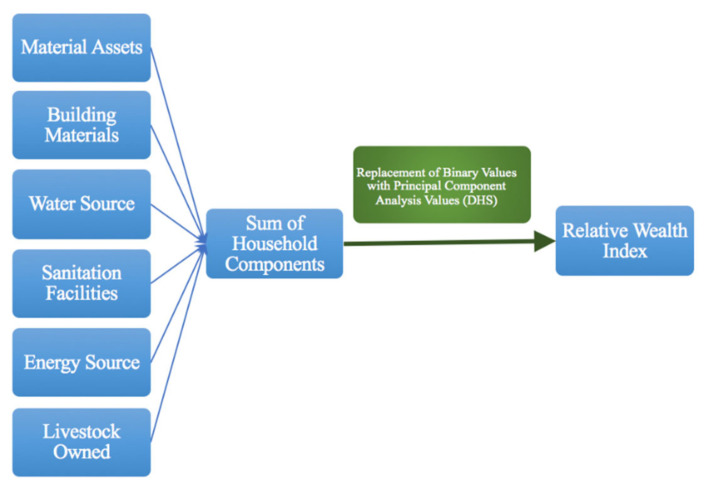
Flowchart of relative wealth index construction.

To determine whether intracommunity wealth differences are related to water access we chose to explore the socioeconomic information and ‘walk time to water source’ variables as the exposure and outcome variables, respectively. Walk time to water was self-reported by respondents as part of the administered household survey. Overall socioeconomic status, assessed as ‘relative wealth’, was determined through the construction of a wealth index so that the socioeconomic variation between households within the Bolde community could be assessed. Many public health studies have indicated that a wealth index is a fair proxy for household wealth when no data on household income is available [22,23,24]. This was the case of this particular community, as the majority of village residents worked as subsistence farmers and thus did not have a formal, standard, easily measurable quantitative income. Creating a wealth index made it possible to observe the relative socioeconomic position of households within a particular community, while also allowing us to recognize the specific needs of rural communities as opposed to their urban counterparts [23]. In terms of the outcome variable, time to obtain water was used as an indicator of household water access, in accordance with the United Nation’s Sustainable Development Goals’ inclusion of “walking time to water” as a crucial contributor to water accessibility [3]. This decision is also based on the knowledge that the time necessary to reach a household’s given water source has direct impacts on the quantities of water the water bearer is able to collect and the household’s overall health [24]. There are two relevant methods for wealth index construction available to be used in the context of this project: raw asset sums, or an analytical compilation of various weighted wealth indicators. While calculating the raw sums of material assets would likely have been simpler, it would not comprehensively encapsulate the regionally specific values with respect to particular items [25]. Thus, the wealth index constructed for this study incorporated multiple household components, including personal material assets, building materials, type of household energy source, type of household water source, livestock owned, and household sanitation facilities (Figure 1). This data, when compiled into a single index, provided more comprehensive insights into the relative wealth of a given household within the context of its own community. 

All relevant wealth data were first deconstructed into binary codes (0 if the household did not have a particular item, and 1 for if it had an item) for each particular asset (See Table 1). 

Of course, certain types of assets are inherently worth higher economic value than others. Therefore, we incorporated principal component analysis (PCA) to construct community-relevant weighted values. The PCA method weighs particular items in terms of their relevant value within a single setting, with the least common items having the higher values and more common items having lower values. The combined effect of these weighted items provided a more realistic assessment of a household’s status [26,27]. The PCA values of the specific items included in the wealth index were assigned to designated items and materials based on prior cross-sectional data from the Nepal 2016 Demographic Health Survey (see Supplementary Materials Table S1). This cross-sectional survey data mathematically determined the weights of household items within both urban and rural settings [28]. Once all weights were assigned to the binary output of each respective item, each surveyed household had its index components added together; the weighted sum of each household was considered its relative wealth index. All households were numerically ranked based on wealth index and then categorically divided into quintiles for further analysis.

The wealth index took the form of a normal distribution, with a minimum, maximum, mean, and standard deviation of −0.63, 1.21, −0.12, and 0.37, respectively, indicating heterogeneity (or an adequate range in wealth in the community to assess this characteristic in relation to water access). A correlation matrix (scaled −1 to 1) was generated for the wealth index, to determine whether any variables should be dropped. The World Food Program’s Vulnerability Assessment and Mapping Guidance Paper on wealth index construction indicates that investigators should use a correlation threshold of 0.9 when determining whether to remove variables from the index [29]. We elected to use an even more conservative threshold of 0.8. Our correlation matrix did not indicate that any variables were highly correlated and warrant removal from the wealth index.Both relative wealth and water collection times were then designated into binary categorical bins. For the purposes of this analysis, the binary was designated across the division between the second and third quintiles. In other words, the surveyed Bolde households that fell within the bottom 40% of the ranked relative wealth indices were assigned to the low relative wealth category, and all others assigned to the non-low wealth category.

For water retrieval time, a threshold of 30 min was chosen. This was determined in accordance with the UN Sustainable Development Goals [3]. With the binary dependent and independent variables in place, RStudio (R Foundation for Statistical Computing, Vienna, Austria) was used to run a Fisher’s exact test of independence to investigate a potential relationship between low relative wealth within a given rural community and access to water as designated by water retrieval time. In order to account for potential confounding between the “water source” wealth index component and our outcome variable (water retrieval time), we removed the “water source” factor from the wealth index when performing the statistical analysis. The “water source” factor remained as part of the comprehensive wealth index for use in the geospatial analysis of our project.

### 2.3. Geospatial Suitability Analysis

We employed a geospatial information system (GIS) to determine optimal locations for a future public tap to be built in the Bolde region. When household surveys were conducted, the location of each household was collected via the internal GPS on the devise used to collect answers to the survey. This location data were uploaded into ArcGIS Online (ESRI, Redlands, CA, USA) to determine suitable sites for future construction of a community well to lessen local water access barriers.

Three location characteristics were included in the suitability analysis: (1) walking distance to water, (2) current household with existing private or shared taps, and (3) relative wealth (based on the index described above). Each factor was used to create an individual map layer, which were then combined (overlaid) to determine regions of overlap where all criteria were satisfied. 

Within the context of the SDG’s, an inclusive 30 min threshold of round-trip walking distance to water was used as the first layer. The 72 households surveyed were divided into the same binary categories as used in the statistical analysis, and only households that had travel times greater than 30 min were included in this layer. Once these locations were uploaded to ArcGIS, we estimated the area that could be reachable within 15 min (to fit the 30 min round trip standard). The program took into account proximity to existing roads and walkways as well as slope to provide locally informed and accurate estimates for travel time.

The second criterion incorporated into the geospatial analysis was that households without access to a private courtyard or shared tap be prioritized. To do this, the general location data was filtered by household water source; those that listed “private tap” or “shared tap with neighbors” were excluded from this map layer. The remaining households were then organized into location-based clusters to find the most central and easily accessible areas. We created a buffer area of 200 m around the clusters. This ensured that those households that currently did not have immediately available taps on the premises would have access to new source.

The final criteria addressed the environmental justice implications of the impact of household wealth on water access. To reduce any socioeconomic and public health inequalities within the community of interest, a third layer was added to introduce the wealth index data (described above) into the mapping analysis. The location data was stratified by wealth index quintile, with only the lowest 40% included in this map layer so that the poorest community members would be prioritized and included in the consideration of the suitable sites of new source construction. The ArcGIS software’s proximity analysis tool was used to determine the area reachable within a 10 min round trip walk time. A 10 min threshold was chosen to tighten the bounds of optimal locations, as a 30 min round trip threshold for this layer was not specific enough to allow for the successful identification of particular sites.

Once all three individual map layers were constructed, they were combined to determine potential locations for the construction of a new well. Therefore, the new well would cater to households that currently had much longer water collection times, did not have a tap in close proximity, and belonged to the poorest members of the community. 

## 3. Results

### 3.1. Fisher’s Analysis

The Fisher’s exact test of independence did not indicate that the households belonging to the lowest relative wealth quintiles were disproportionately affected by having longer water retrieval walk times (*p* > 0.99). The large *p*-value returned by the statistical analysis is likely due to the small sample size (*n* = 72).

### 3.2. Geospatial Suitability Analysis

**Figure 2:**
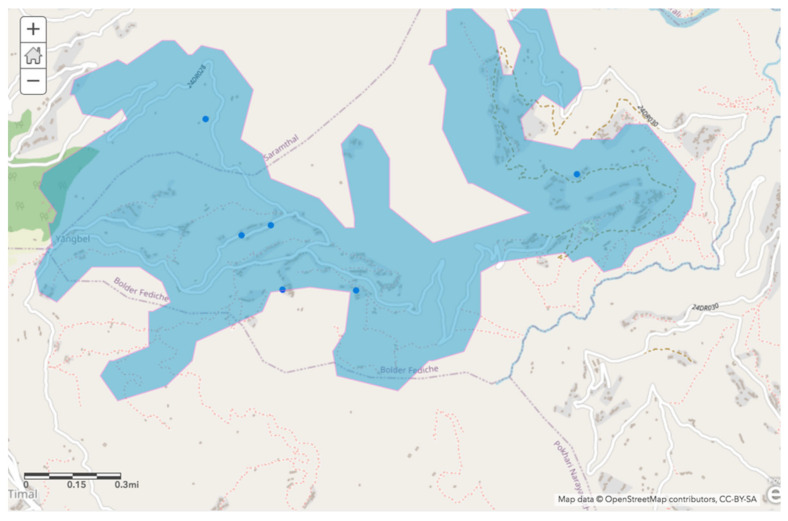
Bolde households with greater than 30 min round-trip walk times to their improved water source (blue points) and area reachable within 30 min (highlighted region).

The three geospatial analysis layers—walking distance to water, current tap availability, and walking distance from households within the lowest quintiles of relative wealth—allowed us to narrow down the geographical scope of ideal water source installation. The first layered analysis displays which regions of Bolde are accessible within a 30 min roundtrip walk time (Figure 2). The starting points for this layer were all households that did not meet SDG guidelines for adequate water access.

**Figure 3:**
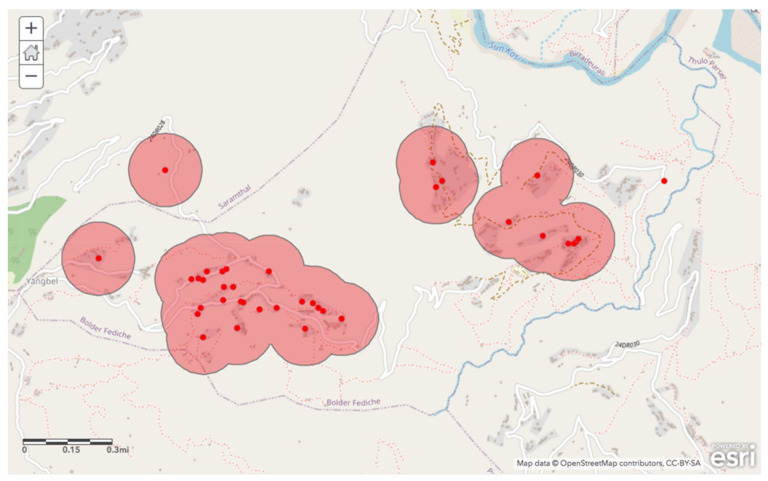
Sampled households without access to a tap on immediate premises (red points), and area within 200 m of household clusters (highlighted region).

The second layer indicates prioritized regions based on areas where households do not already have easy access to a private tap or shared tap among neighbors (Figure 3). The highlighted range illustrates a 200 m radius around centralized clusters of these households. These households are indicated by red points on the map.

**Figure 4:**
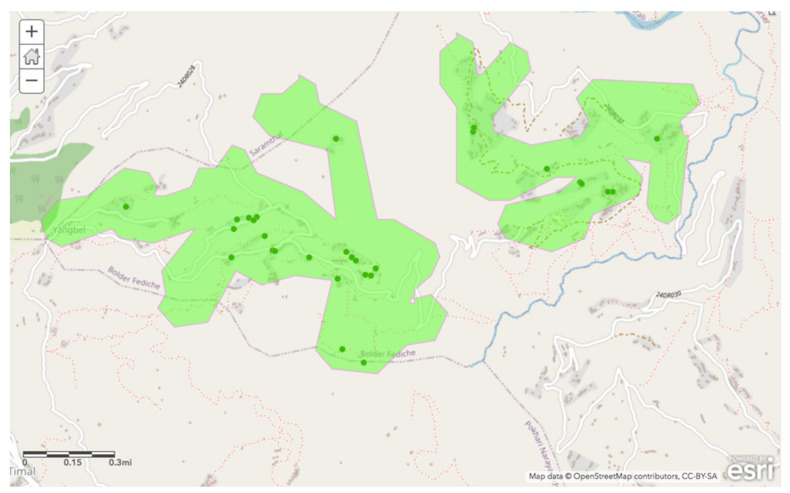
Sampled households that fall within the bottom two quintiles of relative wealth (green points), and area reachable within a 10 min round trip (highlighted region)**.**

Finally, the third layer incorporated a 10 min round trip walking distance from households within the bottom relative wealth quintile (Figure 4). A shorter travel time was selected for this layer in order to remediate socioeconomic inequalities based on studies noted in the literature review [9,14,15,20].

**Figure 5:**
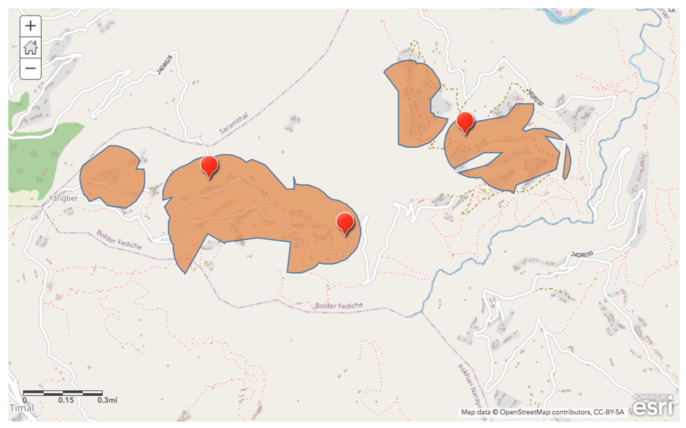
Suitable regions for improved water source construction sites based on superimposed layers of water access, available taps, and relative wealth factors, with recommendations for three optimal sites (red marked locations).

Areas that satisfied all three criteria are shown in Figure 5. Specific points of three ideal tap construction locations were suggested to maximize access for all especially vulnerable households as determined by the study.

## 4. Discussion

Based on the Fisher’s exact test, there was no evidence of an association between wealth and access to water. This lack of association was surprising due to knowledge about the level of water inaccessibility in the area, as well as surveyor anecdotal evidence on the socioeconomic heterogeneity among households in the village of Bolde. This result may be attributed to the small sample size of village households included in the study. However, to prevent the further isolation of the most economically vulnerable (and the body of evidence indicating socioeconomic status as a confounder for water access), these households were still prioritized in the geospatial suitability analysis for future public tap installation. Our finding also contradicts the trends put forth by prior studies assessing the impacts of socioeconomic status on water access at the intercommunity, national, and global level [14,15,20] likely for the same reasons. 

Other studies have constructed wealth indices based on income, but in cases where ‘income’ is hard to assess (such as for members of our study population, who are primarily subsistence farmers), indices are commonly constructed using household assets. There are two commonly used methods of wealth index construction based in asset possession: raw asset sums or weighted asset sums. We chose to use the latter because of its relevance in a given setting, in our case a rural Nepali context; raw asset sums do not necessarily take regional context into account, and thus would not be as useful in a community case study. Moreover, this method will be more easily replicated by future community-based studies due to localized asset data publicly available through the Demographic Health Survey. It is possible that our unexpected results could be attributed to the method of wealth assessment. Based on work by others, the choice was made to use prior national principal component analysis data to provide weighted measures for comprehensive contributors to overall household wealth [26]. However, other options for wealth index construction, such as raw sums of personal assets, may have been less expensive and easier to construct and analyze [25]. Doing so may have led to different overall results indicating a potentially different relationship. Even with the unexpected result provided by the data analysis segment of the study, this finding did not inhibit the spatial analysis approach.

The most prominent strength of this study was the focus on and inclusion of the community members. One way that this was realized was through the continuity of the individuals included in the study sample. The same school children (grades 6−8) included in the 2018 anthropometric study were included in the 2019 household surveys (now in grades 7−9). This not only allowed for consistency in terms of maintaining a single subject population but also showed the participants that their participation was valued and central to the WASH development of their community. Community trust was also prioritized through communication with and input from village leaders, such as the guidance and consent of the secondary school principals. Additionally, the reliance on word-of-mouth directions from neighbors as well as the hiring of Nepali data collectors and translators helped to ensure that community members felt valued, safe, and supported when participating in the household surveys. We used the results of the wealth-based analysis, as well as other known characteristics regarding household water access, to provide a forward-thinking possible plan of action to isolate ideal locations for improved water sources, maximizing accessibility for those most disadvantaged within the community.

The primary challenge that remains at the conclusion of this study is that steps will be taken to ensure that the most sustainable and community-endorsed infrastructure, (i.e., improved water sources), will be incorporated into development plans. This study has identified ideal locations on the basis of individual household characteristics and wealth data, but some questions remain. For example, future development must consider what type of improved water source is most ideal at each location—a public tap or standpipe, a tube well or borehole, or a protected dug well. This decision will likely rely on community preferences, hydrology, physical feasibility, and building costs. Specifically, researchers and rural development teams should seek community input for the final site of construction through partnerships with Village Development Council leadership. The Bolde VDC will be able to provide feedback to the community and insights regarding ideal sites for future wells with the help of the geospatial analyses and maps generated in this study. There will also be a need to ensure that any public improved water source addresses remaining inequities between those households that utilize it, for example, ensuring that social status within the community does not garner greater access to a new source.

## 5. Conclusions

While wide-spread studies have previously been conducted to indicate the inherent differences in WASH accessibility between regions, this study uniquely addressed the implications of water access within a single community in rural Nepal. Our community case study revealed the value of a targeted approach to water inaccessibility in the context of relative wealth and successfully used geospatial analysis tools to indicate practical solutions for the community. In addition, we hope that the methods of this research (wealth index construction in a rural community, mapping, and targeted intervention) can be utilized by researchers in the future. Overall, we point to a greater need for research at the community level as well as efforts to shift the public health research paradigm to include more locally informed, action-oriented approaches to respond to community needs.

## Figures and Tables

**Table 1:** Individual items of the relative wealth index and their respective PCA values (derived from the 2016 Nepal Demographic Health Survey).

Household Component Category	Item	Principal Component Analysis Value	
		If Has, Add:	If Does Not Have, Add:
Material Assets	Radio	0.003	−0.001
	Television	0.103	−0.049
	Mobile Phone	0.011	−0.093
	Table	0.099	−0.067
	Cupboard	0.091	−0.045
	Watch	0.017	−0.028
	Bike	0.111	−0.042
	Motorbike	0.196	−0.020
	Car/truck	0.209	−0.004
Building Materials *Roof* *Wall* *Floor*	Corrugated iron	0.001	-0.001
	Tiles	−0.021	0.010
	Concrete	0.207	−0.026
	Stone	−0.103	0.001
	Brick	0.113	−0.009
	Mud	0.008	−0.001
	Iron	−0.042	0.002
	Cement	0.207	−0.026
	Mud	−0.043	0.135
	Cement	0.175	−0.031
	Soil/Sand	−0.044	0.135
Energy Source	Wood	−0.036	0.142
	Liquid Propane Gas	0.201	−0.019
	Coal	−0.005	4.970 × 10^−6^
Water Source	Private tap in court	−0.028	0.011
	Shared tap with neighbors	−0.056	0.001
	Public tap	−0.074	0.029
	Borehole	0.099	−0.047
	Surface water	−0.099	0.002
Sanitation Facilities	Water seal latrine	−0.070	0.013
	Pit latrine	−0.058	0.001
	Pit slab latrine	−0.083	0.004
	No latrine	0.007	−0.001
Livestock Owned	Goat	0.001	−0.001
	Buffalo	−0.020	0.013
	Poultry	−0.047	0.026
	Cow	−0.026	0.023

## Supplementary Materials

The following are available online at https://www.mdpi.com/1660-4601/17/18/6517/s1, Table S1: DHS Principal Component Analysis Values of Household Assets for Wealth Index Construction in Rural, Nepal DHS 2016.
