# Supplementary material for: Assessing the Impacts of Relative Wealth and Geospatial Factors on Water Access in Rural Nepal: A Community Case Study
Source: Int J Environ Res Public Health. 2020 Sep 7;17(18):6517. doi: 10.3390/ijerph17186517 (PMC7559425; doi:10.3390/ijerph17186517)
Supplement: Supplementary file 1 [file ijerph-17-06517-s001.pdf]

upplementary Table 1. DHS Principal Component Analysis Values of Household Assets for Wealth Index Construction in Rural Nepal [1].

Descriptive Statistics					Component Score Coefficient Matrix		Sum over each variable	
						Component		
	Mean	Std. Deviation [a]	Analysis N[a]	Missing N		1	If has	If does not have
QH101_11 Source of drinking water: Piped into dwelling	0.02	0.132	4062	0	QH101_11 Source of drinking water: Piped into dwelling	0.025	0.188755072	−0.003406107
QH101_12 Source of drinking water: Piped to yard/plot	0.28	0.450	4062	0	QH101_12 Source of drinking water: Piped to yard/plot	−0.017	−0.027776782	0.010903125
QH101_13 Source of drinking water: Piped to neighbor	0.02	0.144	4062	0	QH101_13 Source of drinking water: Piped to neighbor	−0.008	−0.055778827	0.001206484
QH101_14 Source of drinking water: Public tap/standpipe	0.28	0.449	4062	0	QH101_14 Source of drinking water: Public tap/standpipe	−0.046	−0.074298675	0.029022522
QH101_21 Source of drinking water: Tube well or borehole	0.32	0.468	4062	0	QH101_21 Source of drinking water: Tube well or borehole	0.069	0.098823098	−0.047573482
QH101_31 Source of drinking water: Protected well	0.01	0.072	4062	0	QH101_31 Source of drinking water: Protected well	0.001	0.012255179	−6.36869E-05
QH101_32 Source of drinking water: Unprotected well	0.00	0.066	4062	0	QH101_32 Source of drinking water: Unprotected well	−0.001	−0.020758336	9.23962E-05
QH101_41 Source of drinking water: Protected spring	0.02	0.125	4062	0	QH101_41 Source of drinking water: Protected spring	−0.009	−0.067379555	0.00109574
QH101_42 Source of drinking water: Unprotected spring	0.02	0.146	4062	0	QH101_42 Source of drinking water: Unprotected spring	−0.016	−0.10436219	0.002337839
QH101_81 Source of drinking water: Surface water (river/dam/lake/pond/stream/canal/irrigation channel)	0.02	0.155	4062	0	QH101_81 Source of drinking water: Surface water (river/dam/lake/pond/stream/canal/irrigation channel)	−0.016	−0.098954818	0.002497598
QH101_91 Source of drinking water: Bottled water	0.00	0.031	4062	0	QH101_91 Source of drinking water: Bottled water	0.009	0.28365642	−0.000279602
QH101_96 Source of drinking water: Other	0.00	0.016	4062	0	QH101_96 Source of drinking water: Other	−0.002	−0.11561814	2.84704E-05
QH109_11 Type of toilet facility: Flush to piped sewer system	0.00	0.054	4062	0	QH109_11 Type of toilet facility: Flush to piped sewer system	0.004	0.070597387	−0.000209177
QH109_12 Type of toilet facility: Flush to septic tank	0.41	0.492	4062	0	QH109_12 Type of toilet facility: Flush to septic tank	0.019	0.022712773	−0.015857161
QH109_13 Type of toilet facility: Flush to pit latrine	0.16	0.366	4062	0	QH109_13 Type of toilet facility: Flush to pit latrine	−0.030	−0.069834562	0.013279411
QH109_14 Type of toilet facility: Flush to somewhere else	0.00	0.052	4062	0	QH109_14 Type of toilet facility: Flush to somewhere else	−0.004	−0.071042074	0.000192906
QH109_21 Type of toilet facility:	0.04	0.199	4062	0	QH109_21 Type of toilet facility:	0.013	0.061982139	−0.002657514

Ventilated improved pit latrine					Ventilated improved pit latrine			
QH109_22 Type of toilet facility: Pit latrine with slab	0.05	0.211	4062	0	QH109_22 Type of toilet facility: Pit latrine with slab	−0.018	−0.082652417	0.004033387
QH109_23 Type of toilet facility: Pit latrine without slab/open pit	0.01	0.094	4062	0	QH109_23 Type of toilet facility: Pit latrine without slab/open pit	−0.005	−0.057905386	0.000517783
QH109_31 Type of toilet facility: Composting toilet	0.01	0.098	4062	0	QH109_31 Type of toilet facility: Composting toilet	0.013	0.132197936	−0.001281561
QH109_61 Type of toilet facility: No facility/bush/field	0.16	0.369	4062	0	QH109_61 Type of toilet facility: No facility/bush/field	0.003	0.007561751	−0.001472317
QH109_11_sh Type of toilet facility: Flush to piped sewer system - shared	0.00	0.038	4062	0	QH109_11_sh Type of toilet facility: Flush to piped sewer system - shared	0.010	0.260308018	−0.000385071
QH109_12_sh Type of toilet facility: Flush to septic tank - shared	0.09	0.282	4062	0	QH109_12_sh Type of toilet facility: Flush to septic tank - shared	0.006	0.020072353	−0.001916293
QH109_13_sh Type of toilet facility: Flush to pit latrine - shared	0.04	0.203	4062	0	QH109_13_sh Type of toilet facility: Flush to pit latrine - shared	−0.012	−0.054618603	0.002444351
QH109_14_sh Type of toilet facility: Flush to somewhere else - shared	0.00	0.016	4062	0	QH109_14_sh Type of toilet facility: Flush to somewhere else - shared	−0.001	−0.078880239	1.94238E-05
QH109_21_sh Type of toilet facility: Ventilated improved pit latrine - shared	0.01	0.099	4062	0	QH109_21_sh Type of toilet facility: Ventilated improved pit latrine - shared	0.010	0.098615036	−0.000980756
QH109_22_sh Type of toilet facility: Pit latrine with slab - shared	0.00	0.065	4062	0	QH109_22_sh Type of toilet facility: Pit latrine with slab - shared	−0.002	−0.028108102	0.00011813
QH109_23_sh Type of toilet facility: Pit latrine without slab/open pit - shared	0.00	0.044	4062	0	QH109_23_sh Type of toilet facility: Pit latrine without slab/open pit - shared	−0.002	−0.047743831	9.42157E-05
QH109_31_sh Type of toilet facility: Composting toilet - shared	0.01	0.078	4062	0	QH109_31_sh Type of toilet facility: Composting toilet - shared	0.008	0.103267775	−0.000639508
QH113_1 Type of cooking fuel: Electricity	0.01	0.075	4062	0	QH113_1 Type of cooking fuel: Electricity	0.003	0.043992085	−0.000250512
QH113_2 Type of cooking fuel: LPG	0.09	0.284	4062	0	QH113_2 Type of cooking fuel: LPG	0.062	0.200527395	−0.019381428
QH113_4 Type of cooking fuel: Biogas	0.02	0.136	4062	0	QH113_4 Type of cooking fuel: Biogas	0.022	0.160443941	−0.003100172
QH113_5 Type of cooking fuel: Kerosene	0.00	0.016	4062	0	QH113_5 Type of cooking fuel: Kerosene	−0.001	−0.072422858	1.78337E-05
QH113_7 Type of cooking fuel: Charcoal	0.00	0.031	4062	0	QH113_7 Type of cooking fuel: Charcoal	0.000	−0.005042541	4.97047E-06
QH113_8 Type of cooking fuel: Wood	0.80	0.398	4062	0	QH113_8 Type of cooking fuel: Wood	−0.073	−0.036282951	0.148174303
QH113_9 Type of cooking fuel: Straw/shrubs/grass	0.02	0.150	4062	0	QH113_9 Type of cooking fuel: Straw/shrubs/grass	0.010	0.064149678	−0.001503129
QH113_10 Type of cooking fuel: Agricultural crop	0.01	0.073	4062	0	QH113_10 Type of cooking fuel: Agricultural crop	0.004	0.051190885	−0.000278762
QH113_11 Type of cooking fuel: Animal dung	0.05	0.225	4062	0	QH113_11 Type of cooking fuel: Animal dung	0.029	0.120359967	−0.006825825

QH113_95 Type of cooking fuel: No food cooked in household	0.00	0.027	4062	0	QH113_95 Type of cooking fuel: No food cooked in household	0.000	−0.00051334	3.79409E-07
QH121A Electricity	0.83	0.378	4062	0	QH121A Electricity	0.044	0.020246227	−0.097072137
QH121B Radio	0.31	0.464	4062	0	QH121B Radio	0.002	0.002602304	−0.001190505
QH121C Television	0.32	0.468	4062	0	QH121C Television	0.071	0.103028381	−0.049320102
QH121D Telephone (non-mobile)	0.02	0.136	4062	0	QH121D Telephone (non-mobile)	0.025	0.177889893	−0.00343727
QH121E Computer	0.04	0.200	4062	0	QH121E Computer	0.038	0.182057328	−0.007952144
QH121F Refrigerator	0.05	0.213	4062	0	QH121F Refrigerator	0.057	0.256213812	−0.012780891
QH121G Table	0.40	0.491	4062	0	QH121G Table	0.082	0.099186696	−0.067093271
QH121H Chair	0.43	0.496	4062	0	QH121H Chair	0.082	0.093457083	−0.071452852
QH121I Bed	0.90	0.297	4062	0	QH121I Bed	0.041	0.013337888	−0.123131884
QH121J Sofa	0.06	0.246	4062	0	QH121J Sofa	0.055	0.211265335	−0.014566189
QH121K Cupboard	0.33	0.471	4062	0	QH121K Cupboard	0.064	0.090937701	−0.045267661
QH121L Clock	0.27	0.446	4062	0	QH121L Clock	0.060	0.097725024	−0.036791766
QH121M Fan	0.30	0.457	4062	0	QH121M Fan	0.088	0.135956928	−0.05713994
QH121N Invertor	0.03	0.182	4062	0	QH121N Invertor	0.036	0.19135394	−0.006780066
QH121O Dhiki/Janto	0.48	0.500	4062	0	QH121O Dhiki/Janto	−0.014	−0.014334338	0.013156729
QH122A Watch	0.62	0.486	4062	0	QH122A Watch	0.022	0.017200243	−0.027875491
QH122B Mobile telephone	0.90	0.302	4062	0	QH122B Mobile telephone	0.031	0.010562721	−0.093577506
QH122C Bicycle/ Ricksaw	0.28	0.448	4062	0	QH122C Bicycle/ Ricksaw	0.069	0.110709043	−0.042458577
QH122D Motorcycle or scooter	0.09	0.292	4062	0	QH122D Motorcycle or scooter	0.063	0.196313466	−0.020319324
QH122E Animal-drawn cart	0.03	0.182	4062	0	QH122E Animal-drawn cart	0.023	0.120546959	−0.004271228
QH122F A Car/Truck/Tractor	0.02	0.130	4062	0	QH122F A Car/Truck/Tractor	0.028	0.209536749	−0.003674242
QH122G Three wheeler Tempo	0.00	0.052	4062	0	QH122G Three wheeler Tempo	0.003	0.055274526	−0.000150091
QH123 Bank account	0.59	0.492	4062	0	QH123 Bank account	0.045	0.037958163	−0.054313566
QH142_11 Main floor material: Earth/sand	0.76	0.429	4062	0	QH142_11 Main floor material: Earth/sand	−0.076	−0.04341801	0.134727402
QH142_12 Main floor material: Dung	0.07	0.254	4062	0	QH142_12 Main floor material: Dung	0.014	0.050880068	−0.003795815
QH142_21 Main floor material: Wood planks	0.01	0.088	4062	0	QH142_21 Main floor material: Wood planks	−0.003	−0.029244127	0.000232211
QH142_31 Main floor material: Parquet or polished wood	0.00	0.041	4062	0	QH142_31 Main floor material: Parquet or polished wood	0.001	0.024626565	−4.25119E-05
QH142_33 Main floor material: Ceramic tiles	0.00	0.022	4062	0	QH142_33 Main floor material: Ceramic tiles	0.008	0.379081623	−0.00018674
QH142_34 Main floor material: Cement	0.15	0.359	4062	0	QH142_34 Main floor material: Cement	0.074	0.174603069	−0.031331213

QH142_35 Main floor material: Carpet	0.01	0.099	4062	0
QH142_96 Main floor material: Other	0.00	0.047	4062	0
QH143_12 Main roof material: Thatch/palm leaf	0.14	0.349	4062	0
QH143_13 Main roof material: Mud	0.03	0.182	4062	0
QH143_21 Main roof material: Rustic mat	0.00	0.035	4062	0
QH143_22 Main roof material: Palm/bamboo	0.00	0.066	4062	0
QH143_23 Main roof material: Wood planks	0.01	0.072	4062	0
QH143_31 Main roof material: Metal	0.37	0.482	4062	0
QH143_32 Main roof material: Wood	0.00	0.038	4062	0
QH143_33 Main roof material: Calamine/cement fiber	0.01	0.095	4062	0
QH143_34 Main roof material: Ceramic tiles	0.32	0.467	4062	0
QH143_35 Main roof material: Cement	0.11	0.313	4062	0
QH143_36 Main roof material: Roofing shingles	0.00	0.041	4062	0
QH143_96 Main roof material: Other	0.00	0.035	4062	0
QH144_12 Main wall material: Cane/palm/trunks	0.01	0.095	4062	0
QH144_13 Main wall material: Mud/Sand	0.08	0.272	4062	0
QH144_21 Main wall material: Bamboo with mud	0.15	0.359	4062	0
QH144_22 Main wall material: Stone with mud	0.46	0.498	4062	0
QH144_23 Main wall material: Plywood	0.00	0.027	4062	0
QH144_24 Main wall material: Cardboard	0.00	0.022	4062	0
QH144_25 Main wall material: Reused wood	0.01	0.084	4062	0
QH144_26 Main wall material: Metal/Galvanized sheet	0.05	0.210	4062	0
QH144_31 Main wall material: Cement	0.14	0.348	4062	0
QH144_32 Main wall material: Stone with lime/cement	0.01	0.119	4062	0
QH144_33 Main wall material: Bricks	0.07	0.257	4062	0
QH144_34 Main wall material: Cement blocks	0.00	0.061	4062	0

QH142_35 Main floor material: Carpet	0.029
QH142_96 Main floor material: Other	−0.003
QH143_12 Main roof material: Thatch/palm leaf	−0.034
QH143_13 Main roof material: Mud	−0.025
QH143_21 Main roof material: Rustic mat	−0.003
QH143_22 Main roof material: Palm/bamboo	−0.005
QH143_23 Main roof material: Wood planks	−0.005
QH143_31 Main roof material: Metal	0.001
QH143_32 Main roof material: Wood	−0.002
QH143_33 Main roof material: Calamine/cement fiber	0.009
QH143_34 Main roof material: Ceramic tiles	−0.015
QH143_35 Main roof material: Cement	0.073
QH143_36 Main roof material: Roofing shingles	−0.002
QH143_96 Main roof material: Other	−0.004
QH144_12 Main wall material: Cane/palm/trunks	−0.003
QH144_13 Main wall material: Mud/Sand	0.002
QH144_21 Main wall material: Bamboo with mud	0.016
QH144_22 Main wall material: Stone with mud	−0.078
QH144_23 Main wall material: Plywood	0.000
QH144_24 Main wall material: Cardboard	0.001
QH144_25 Main wall material: Reused wood	−0.005
QH144_26 Main wall material: Metal/Galvanized sheet	−0.009
QH144_31 Main wall material: Cement	0.074
QH144_32 Main wall material: Stone with lime/cement	0.006
QH144_33 Main wall material: Bricks	0.031
QH144_34 Main wall material: Cement blocks	0.007

0.292110445	−0.002905126
−0.061322476	0.000136171
−0.082993582	0.013713225
−0.132008333	0.004677328
−0.097338949	0.000119964
−0.077011652	0.000342782
−0.075882133	0.000394339
0.001302007	−0.000759081
−0.051265838	7.5837E-05
0.095172397	−0.000874877
−0.021085516	0.00998063
0.207580809	−0.025667668
−0.042338044	7.30866E-05
−0.103470484	0.000127521
−0.028435665	0.000261396
0.008395401	−0.000737464
0.038885927	−0.00695117
−0.085019335	0.07153032
0.005728167	−4.23368E-06
0.042157899	−2.07674E-05
−0.060931616	0.00043814
−0.041722704	0.002024747
0.183302407	−0.030042687
0.049958832	−0.000723679
0.112821931	−0.008609623
0.115519787	−0.000428168

QH144_35 Main wall material: Wood planks/shingles	0.02	0.128	4062	0	QH144_35 Main wall material: Wood planks/shingles	0.000	−0.002560891	4.36005E-05
QH144_96 Main wall material: Other	0.00	0.022	4062	0	QH144_96 Main wall material: Other	−0.002	−0.073996531	3.64515E-05
HOUSE Owns a house	0.12	0.330	4062	0	HOUSE Owns a house	0.013	0.034958083	−0.004963124
LAND Owns land	0.90	0.298	4062	0	LAND Owns land	−0.019	−0.006400254	0.058594321
memsleep Number of members per sleeping room	2.15	1.438	4062	0	memsleep Number of members per sleeping room	−0.025	0.020092919	0.037603634
QH118A_1 Cows/bulls: 1—4	0.4655	0.49887	4062	0	QH118A_1 Cows/bulls: 1—4	−0.024	−0.025868525	0.022532189
QH118A_2 Cows/bulls: 5—9	0.0953	0.29363	4062	0	QH118A_2 Cows/bulls: 5—9	−0.025	−0.076307542	0.008035651
QH118A_3 Cows/bulls: 10+	0.0091	0.09502	4062	0	QH118A_3 Cows/bulls: 10+	−0.008	−0.08338057	0.00076648
QH118B_1 Other cattle - buffalo: 1—4	0.3983	0.48961	4062	0	QH118B_1 Other cattle - buffalo: 1—4	−0.017	−0.020294088	0.013435284
QH118B_2 Other cattle - buffalo: 5—9	0.0098	0.09876	4062	0	QH118B_2 Other cattle - buffalo: 5—9	0.004	0.040509744	−0.000402882
QH118B_3 Other cattle - buffalo: 10+	0.0012	0.03507	4062	0	QH118B_3 Other cattle - buffalo: 10+	0.005	0.143764702	-0.000177181
QH118C_1 Horses/donkeys/mules: 1—4	0.0089	0.09373	4062	0	QH118C_1 Horses/donkeys/mules: 1—4	−0.009	−0.100107807	0.000895152
QH118C_2 Horses/donkeys/mules: 5+	0.0027	0.05197	4062	0	QH118C_2 Horses/donkeys/mules: 5+	−0.005	−0.096332936	0.00026158
QH118D_1 Goats: 1—4	0.4057	0.49109	4062	0	QH118D_1 Goats: 1—4	0.000	0.000565584	−0.000386115
QH118D_2 Goats: 5—9	0.1672	0.37316	4062	0	QH118D_2 Goats: 5—9	−0.011	−0.025237807	0.005065466
QH118D_3 Goats: 10+	0.0662	0.24870	4062	0	QH118D_3 Goats: 10+	−0.019	−0.070699663	0.005014028
QH118E_1 Sheep: 1—4	0.0226	0.14880	4062	0	QH118E_1 Sheep: 1—4	−0.009	−0.058321871	0.00135154
QH118E_2 Sheep: 5—9	0.0049	0.07000	4062	0	QH118E_2 Sheep: 5—9	−0.007	−0.099745613	0.000493546
QH118E_3 Sheep: 10+	0.0086	0.09244	4062	0	QH118E_3 Sheep: 10+	−0.011	−0.12098187	0.001051494
QH118F_1 Chickens or other poultry: 1—9	0.3562	0.47894	4062	0	QH118F_1 Chickens or other poultry: 1—9	−0.035	−0.047379753	0.026217401
QH118F_2 Chickens or other poultry: 10—29	0.1000	0.29997	4062	0	QH118F_2 Chickens or other poultry: 10—29	−0.004	−0.011129989	0.001235989
QH118F_3 Chickens or other poultry: 30+	0.0113	0.10583	4062	0	QH118F_3 Chickens or other poultry: 30+	0.011	0.103769469	−0.001188595

QH118G_1 Ducks: 1—4	0.0249	0.15573	4062	0
QH118G_2 Ducks: 5—9	0.0062	0.07822	4062	0
QH118G_3 Ducks: 10+	0.0027	0.05197	4062	0
QH118H_1 Pigs: 1—4	0.0960	0.29464	4062	0
QH118H_2 Pigs: 5—9	0.0030	0.05428	4062	0
QH118H_3 Pigs: 10+	0.0007	0.02717	4062	0
QH118I_1 Yaks: 1—4	0.0030	0.05428	4062	0
QH118I_2 Yaks: 5—9	0.0012	0.03507	4062	0
QH118I_3 Yaks: 10+	0.0017	0.04148	4062	0
landarea	0.4573	0.74700	4062	21

a. For each variable, missing values are replaced with the variable mean.

QH118G_1 Ducks: 1—4	0.005
QH118G_2 Ducks: 5—9	0.011
QH118G_3 Ducks: 10+	0.007
QH118H_1 Pigs: 1—4	−0.008
QH118H_2 Pigs: 5—9	−0.003
QH118H_3 Pigs: 10+	0.000
QH118I_1 Yaks: 1—4	−0.004
QH118I_2 Yaks: 5—9	−0.002
QH118I_3 Yaks: 10+	−0.005
landarea	0.025

Extraction Method: Principal Component Analysis.
Component Scores.

0.029268409	−0.000746304
0.133703513	−0.000827988
0.138013036	−0.000374758
−0.024436822	0.002595414
−0.053076598	0.000157264
−0.017913421	1.32398E-05
−0.076100748	0.000225484
−0.061475067	7.57642E-05
−0.112162351	0.000193622
0.018449449	−0.015543285

References

1. The DHS Program—Wealth-Index-Construction. Available online: https://www.dhsprogram.com/topics/wealth-index/Wealth-Index-Construction.cfm (accessed on 25 March 2020).
